# Total knee arthroplasty following lateral closing-wedge high tibial osteotomy versus primary total knee arthroplasty: a propensity score matching study

**DOI:** 10.1186/s13018-024-04760-6

**Published:** 2024-05-07

**Authors:** Tianshun Xie, Astrid J. de Vries, Hugo C. van der Veen, Reinoud W. Brouwer

**Affiliations:** 1 Department of Orthopaedic Surgery, University of Groningen, University Medical Center Groningen, P.O. Box 30.001, 9700 RB Groningen, The Netherlands; 2 Department of Orthopaedic Surgery, Martini Hospital, Groningen, The Netherlands

**Keywords:** Total knee arthroplasty, High tibial osteotomy, Patient-reported outcomes, Radiological results, Propensity score match

## Abstract

**Background:**

The disparity in patient-reported outcomes between total knee arthroplasty (TKA) following high tibial osteotomy (HTO) and primary TKA has yet to be fully comprehended. This study aims to compare the patient-reported outcomes, radiological parameters and complication rates between TKA following HTO and primary TKA.

**Methods:**

Sixty-five patients who underwent TKA following lateral closing-wedge HTO were compared to a matched group of primary TKA at postoperative 6-months and 1-year. Between-group confounders of age, gender, smoking status, Body Mass index, preoperative Numeric Rating Scale (NRS) pain in rest, Knee injury and Osteoarthritis Outcome Score-Physical function Shortform (KOOS-PS), EuroQol five-dimensional (EQ-5D) overall health score, and Oxford Knee Score (OKS) were balanced by propensity score matching. Patient-reported outcome measures were NRS pain in rest, KOOS-PS, EQ-5D overall health score, and OKS. Radiological parameters were femorotibial angle, medial proximal tibial angle, anatomical lateral distal femoral angle, posterior tibial slope, and patellar height assessed by Insall-Salvati ratio. The complication rates of TKA were compared between the two groups. The HTO survival time, the choice of staple removal before or during TKA in patients who underwent TKA following HTO patients, and the rate of patellar resurfacing were assessed. The *p* value < 0.0125 indicates statistical significance after Bonferroni correction.

**Results:**

After propensity score matching, no significant between-group differences in the patient-reported outcome measures, radiographical parameters and complication rates were found (*p* > 0.0125). In the TKA following HTO group, with an average HTO survival time of 8.7 years, staples were removed before TKA in 46 patients (71%) and during TKA in 19 patients, and 11 cases (17%) had patella resurfacing. In the primary TKA group, 15 cases (23%) had patella resurfacing.

**Conclusion:**

The short-term assessment of TKA following HTO indicates outcomes similar to primary TKA. A previous HTO does not impact the early results of subsequent TKA, suggesting that the previous HTO has minimal influence on TKA outcomes.

**Level of evidence:**

III, cohort study.

## Introduction

High tibial osteotomy (HTO) has proven to be an effective technique for addressing medial knee osteoarthritis and delivering good clinical outcomes, with 10-years survival rates ranging from 64 to 97.6% and 20-years survival rates ranging from 46 to 85.1% [1]. However, it is important to acknowledge that its efficacy may deteriorate with time [2]. In cases where HTO has failed or in the presence of advanced symptomatic knee osteoarthritis, a total knee arthroplasty (TKA) is performed as the subsequent treatment [3].

Past research has highlighted the technical complexities involved in performing TKA following a previous HTO [4–6], such as the soft tissue balancing and the amount of bone resection at the proximal tibia. Ongoing discussions revolve around whether TKA following HTO yields differing outcomes compared to primary TKA [7–10]. As a result, determining whether a previous HTO can encompass the consequences of TKA may impact the surgeon’s choice for a HTO.

Although previous studies have explored this research topic, there are constraints in comparing clinical results between TKA following HTO and primary TKA [4]. Previous studies mostly relied on the Knee Society Score (KSS) questionnaire [6, 7, 9–15], primarily assessed from the physician’s viewpoint [16, 17]. Using questionnaires that consider the patients’ perspectives is necessary [18, 19]. Previous studies compared patient-reported outcomes (e.g., Western Ontario and McMaster Universities Osteoarthritis Index (WOMAC) and Hospital for Special Surgery (HSS) score) between TKA following HTO and primary TKA with follow-ups ranging from 2 to 13 years, but they did not match preoperative patient-reported outcomes between groups before the comparison [9, 15]. Moreover, the above studies employed a retrospective design with variable postoperative follow-up durations for assessing questionnaire outcomes. To address the ongoing controversy, a study with diverse patient-reported outcome questionnaires, well-matched preoperative patient-reported outcomes, and standardized follow-up durations is needed. Furthermore, the assessment of radiological parameters plays a critical role in confirming the intended alignment post-TKA, and early complication assessment is paramount for ensuring patient safety. Consequently, a comparative analysis of patient-reported outcomes, radiological parameters and complications between TKA following HTO and primary TKA is warranted.

The primary objective of this study is to compare patient-reported outcomes at 6-month and 1-year postoperatively between two groups: patients with TKA following lateral closing-wedge HTO and those with primary TKA. The secondary objectives include comparing radiological parameters and complication rates between these two groups. The hypothesis is that patients with TKA following lateral closing-wedge HTO will present inferior patient-reported outcomes compared to patients with a primary TKA. This hypothesis is based on the fact that HTO alters knee anatomy, affecting alignment and force distribution, impacting joint mechanics and proprioception, potentially leading to inferior patient-reported outcomes [7, 20].

## Materials and methods

### Study design

This cohort study was conducted at a large peripheral hospital in the northern Netherlands, and the patients’ electronic medical records and radiographs were checked. The present study followed the statement of STrengthening the Reporting of OBservational studies in Epidemiology (STROBE) for cohort studies [21]. The ethical committee of our hospital approved this study (MEC no. 2023-105).

### Patients

One-hundred-six patients who had TKA following lateral closing-wedge HTO between January 1, 2016 and December 31, 2022 were screened. Patients were included if they completed the routinely administered questionnaires of Numeric Rating Scale (NRS) pain in rest [22], Knee injury and Osteoarthritis Outcome Score-Physical function Shortform (KOOS-PS) [23], EuroQol 5 Dimension (EQ-5D) overall health score [24], and Oxford Knee Score (OKS) [25] at preoperative, postoperative 6-month and postoperative 1-year, following the LROI (Dutch Arthroplasty Register) protocol. Patients were excluded if they had a previous HTO other than a lateral closing-wedge approach, such as medial opening-wedge HTO, combined wedge osteotomy, or double-level osteotomy.

Three-hundred-one patients who had primary TKA without previous HTO or uni-compartmental knee arthroplasty between January 1, 2019 and December 31, 2022, and completed the above questionnaires at preoperative, postoperative 6-month and postoperative 1-year, were included for matching.

### Surgical treatment

**Fig. 1:**
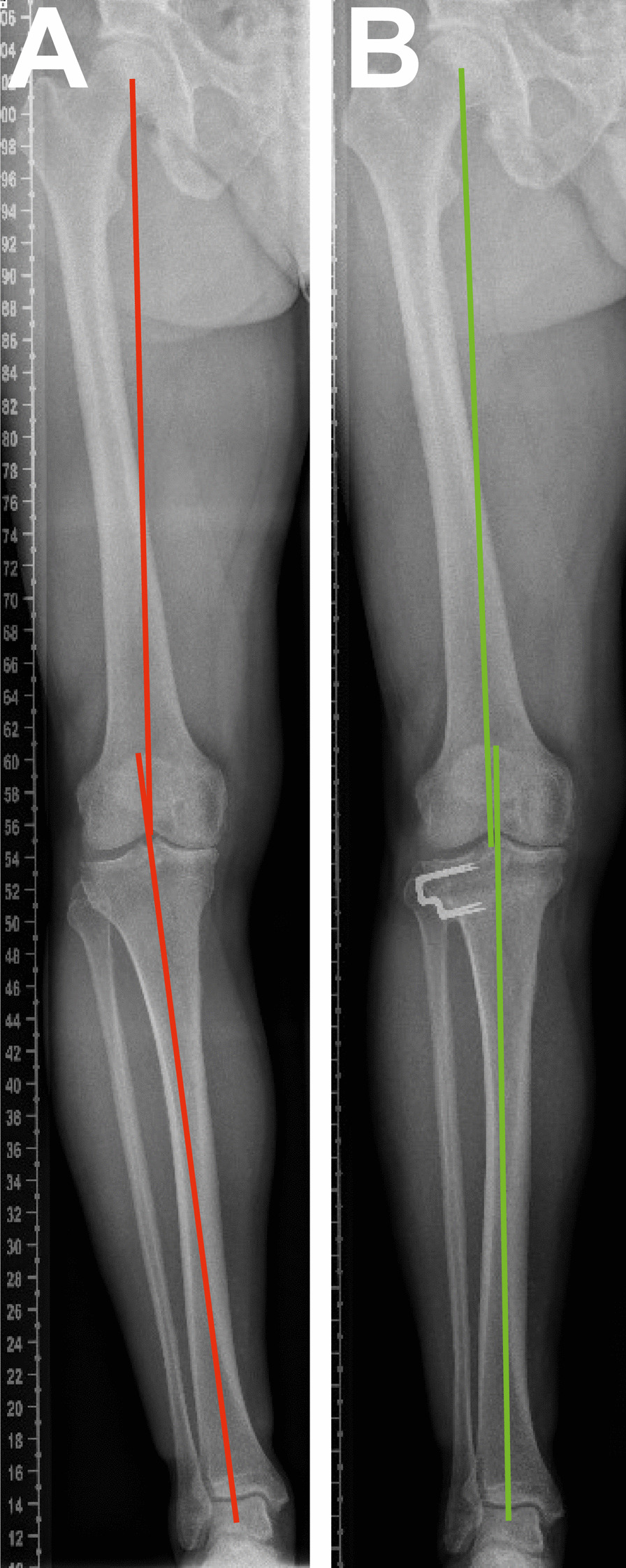
Radiographs taken before and after lateral closing-wedge HTO. *HTO* High tibial osteotomy. **A** Varus mechanical axis before HTO; **B** Valgus mechanical axis after HTO

The surgical technique used for the lateral closing-wedge HTO was performed as described previously [26]. The preoperative planning aimed for a 4° valgus lower limb mechanical axis following HTO [27]. The shift of the lower limb mechanical axis from varus to valgus following a lateral closing-wedge HTO is depicted in Fig. 1.

**Fig. 2:**
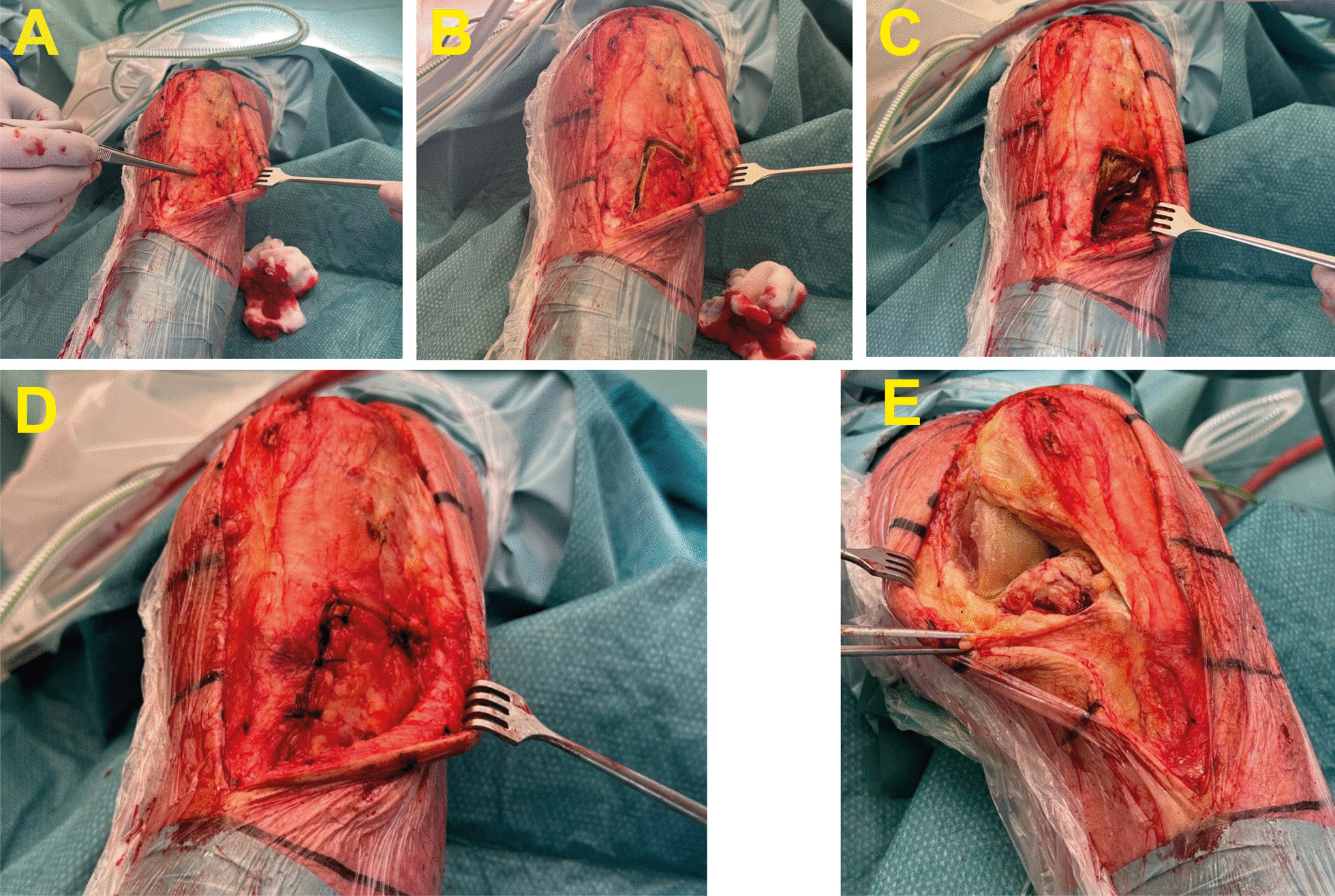
Total knee arthroplasty following lateral closing-wedge high tibial osteotomy. **A** Subcutaneous approach to the anterior compartment; **B** L-incision to the anterior compartment; **C** Release of the staples; **D** Closure after staples removal; **E** Medial approach for total knee arthroplasty

TKA was performed following the standard procedure [28]: the surgical procedure commenced with a midline skin incision, which was succeeded by a medial parapatellar arthrotomy of the joint capsule. In cases of moderate-to-severe patellofemoral osteoarthritis identified, patellar resurfacing was carried out. In this study, both cruciate-retaining or posterior-stabilized TKA implants were used. The photographs of TKA following lateral closing-wedge HTO, including staples removal and tibial osteotomy is illustrated in Fig. 2.

### Patient-reported outcomes

Four questionnaires, NRS pain in rest, KOOS-PS, EQ-5D overall health score and OKS, were routinely administered at three separate time points: preoperative, postoperative 6-months, and postoperative 1-year, following the LROI (Dutch arthroplasty register) protocol.

1. NRS pain in rest [22]: this scale was a generic tool employed to grade pain levels, ranging from 0 to 10, where 1–3 corresponded to mild pain, 4–6 indicated moderate pain, and 7–10 signified severe pain.
2. KOOS-PS [23]: this was a condensed version of KOOS, with scores converted to a 100-point scale. This questionnaire comprises seven items for assessing physical function in knee osteoarthritis. In this study, a score of 0 indicated the highest level of physical function as no difficulty.
3. EQ-5D overall health score [24]: this assessment was employed to evaluate overall health, with scores ranging from 0 to 100. A score of 0 indicated the poorest overall health, while a score of 100 reflected optimal health.
4. OKS [25]: this was utilized to measure pain and function following TKA, ranging from 0 to 48. This questionnaire comprises twelve items. A score of 48 signified the highest level of physical function.

### Radiological parameters

**Fig. 3:**
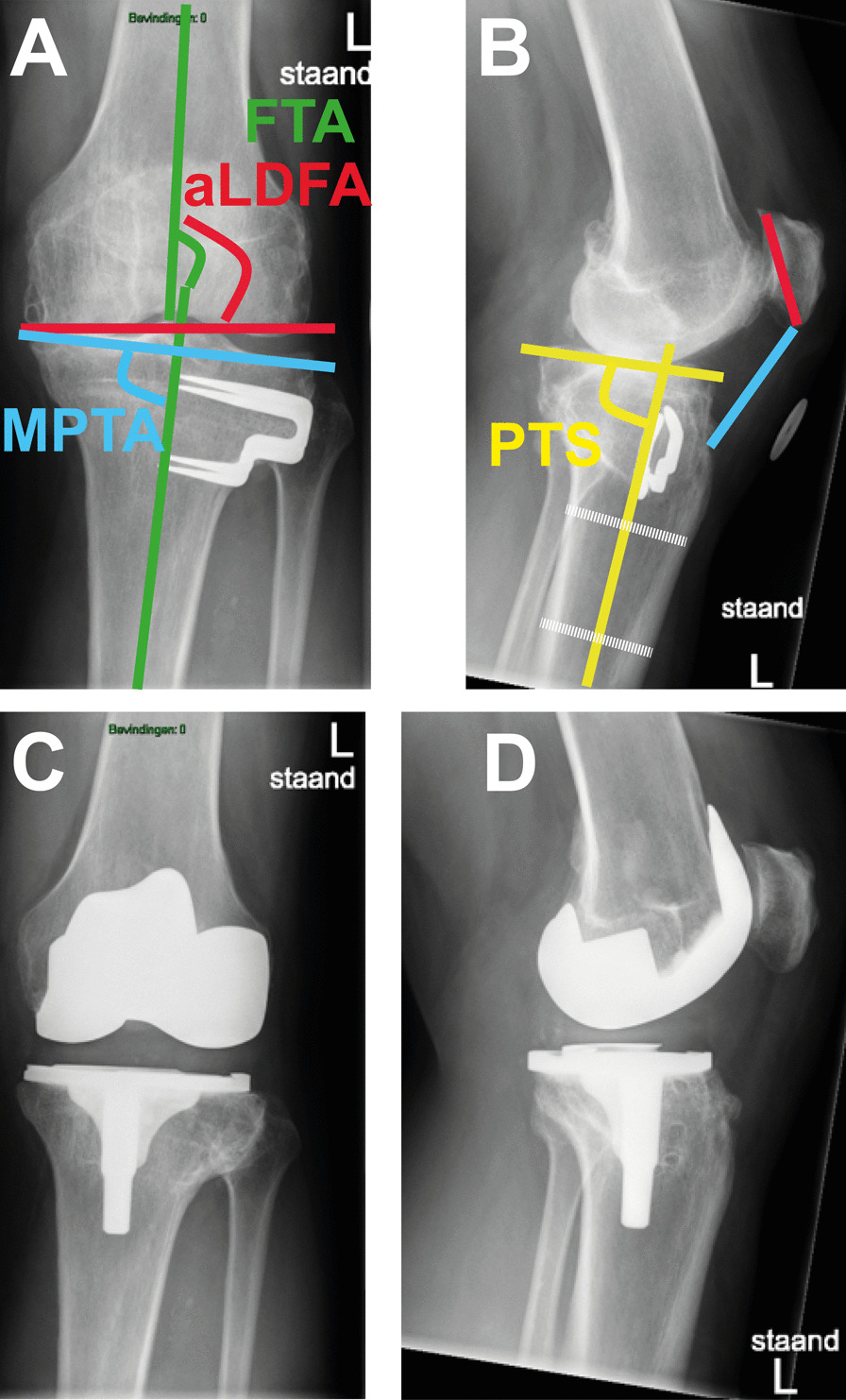
Illustration of radiological measurements. **A** *FTA* Femoral-tibial angle; *aLDFA* anatomical lateral distal femoral angle; *MPTA* medial proximal tibial angle; **B** *PTS* posterior tibial slope; Patellar height via Insall-Salvati method: ratio of blue-line to red-line; **C**, **D** Total knee arthroplasty with Genesis II Posterior Stabilized implant and patellar resurfacing

Radiological parameters, encompassing frontal and sagittal alignments, were assessed. The anteroposterior short-knee standing radiograph was used for measuring knee osteoarthritis grade, and frontal alignments of femoral-tibial angle (FTA), medial proximal tibial angle (MPTA), and anatomical lateral distal femoral angle (aLDFA). The lateral short-knee standing radiograph with 30-degree flexion was used for assessing posterior tibial slope (PTS) and patella height. The radiological parameters are illustrated in Fig. 3.

Kellgren&Lawrence grade [29]: this consisted of four ordinal grades for assessing knee osteoarthritis severity: I (indicating doubtful), II (mild), III (moderate), and IV (severe).

Patellar height: assessed by the Insall-Salvati method [30], which was defined as the ratio of the patellar tendon length to the maximum length of patella in the sagittal plane. The normal range of this ratio was from 0.8 to 1.2.

PTS [31]: the posterior angle between the anatomic axis of the proximal tibia and the tangential line of the medial tibial plateau in the sagittal plane.

FTA [32]: the lateral angle between the distal femoral anatomic axis and proximal tibial anatomic axis in the frontal plane. The normal range for this angle was from 174° to 178°.

MPTA [33, 34]: the medial angle between the anatomical line of proximal tibia and the tangential line of the tibial plateau in the frontal plane. The normal value was 87°, ranging 85°–90°. This angle is also used for assessing knee joint line obliquity [35].

aLDFA [33, 34]: the lateral angle between the distal femoral anatomic axis and the tangential line of the femoral condyles in the frontal plane. The normal value was 81°, ranging 79°–83°.

### Other outcomes

TKA complications in this study were defined as adverse issues arising from TKA that necessitates medical intervention, such as surgical site infection, pulmonary embolism or deep venous thrombosis etc. [36], which were extracted from medical records. In the TKA following HTO group, the HTO survival time was calculated as the duration from the time of HTO to the conversion to TKA in years, and the choice of staple removal before or during TKA in patients who underwent TKA following HTO patients were assessed. The rate of patellar resurfacing during TKA was assessed in each group.

### Propensity score matching

Age [37], gender [38], smoking status [39], and Body Mass index (BMI) at the time of TKA [40] are all factors that impact patient-reported outcomes after TKA. These factors, along with preoperative patient-reported outcome measures including NRS pain in rest, KOOS-PS, EQ-5D overall health score and OKS, were considered confounding variables in this study. They were one-on-one matched between patients undergoing TKA following HTO and those undergoing primary TKA.

### Statistical analysis

SPSS software (version 25) was used for statistical analysis. Propensity score matching method was used with a matching tolerance of 0.02. Distribution of continuous data was checked using Shapiro–Wilk test and Q-Q plot. The independent t tests were used for between-group comparison of parametric continuous data (age at TKA), and Mann-Witney U tests were used for between-group comparison of non-parametric continuous data (BMI at TKA, patient-reported outcome measures, and radiological results). Pearson chi-square tests were used for between-group comparison of gender, smoking status at TKA, and Kellgren&Lawrence grade before TKA. Fisher’s exact tests were used for between-group comparison of TKA complication rate. Continuous data were reported as mean ± standard deviation, and categorical data were presented as numbers and frequencies. The *p*-value < 0.0125 (0.05/4) indicated statistical significance after Bonferroni correction. Based on an effect size of 0.8, a significant level (alpha) of 0.0125, and a study power of 95% determined by the Mann–Whitney U test, a sample size of 58 patients per group was indicated by G*Power software.

## Results

**Fig. 4:**
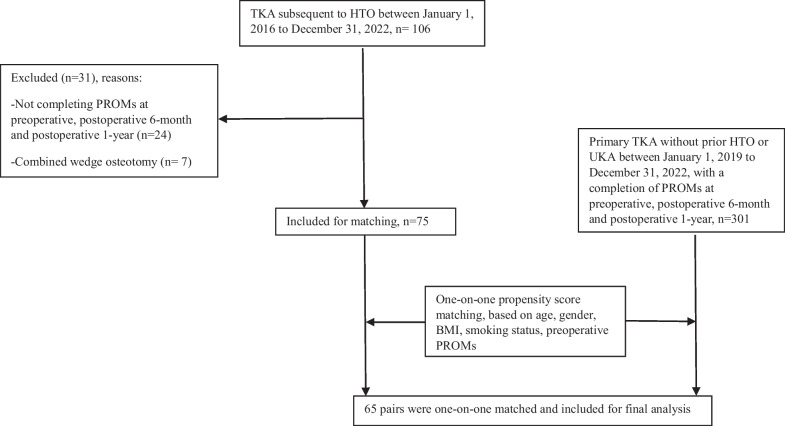
Patient selection process. *TKA* Total knee arthroplasty; *HTO* High tibial osteotomy; *UKA* uni-compartmental knee arthroplasty; *BMI* Body Mass Index; *PROMs* Patient-reported outcome measures. The PROMs are Numeric Rating Scale pain in rest, Knee injury and Osteoarthritis Outcome Score-Physical function Shortform (KOOS-PS), EuroQol 5 Dimension overall health score, and Oxford Knee Score

The patient selection process is shown in Fig. 4. Confounding variables before and after the propensity score matching are presented in Table 1. Three types of primary TKA cemented implants were utilized: GENESIS II Posterior-Stabilized (Smith and Nephew, Memphis, USA), GENESIS II Cruciate-Retaining, and NexGen Legacy® Posterior Stabilized Flex (Zimmer, Warsaw, USA). In TKA following HTO group, the distribution of the above implants was 60/2/2, while in primary TKA, it was 56/6/3. In the TKA following HTO group, one patient received a stemmed tibial component (Legion; Smith and Nephew, Memphis, USA).

**Table 1:** Propensity score matching between groups TKA following HTO and Primary TKA

Confounding variables	Before propensity score matching			After propensity score matching		
	TKA-HTO	Primary TKA	*p* value	TKA-HTO	Primary TKA	*p* value
Number of knees, N	75	301		65	65	
Age at surgery, years	61.8 ± 6.4	70.6 ± 8.5	< 0.001^b^*	62.6 ± 6.0	62.9 ± 6.0	0.782^b^
Gender, male/female	45/30	112/189	< 0.001^c^*	36/29	41/24	0.372^c^
Smoking status, Y/N	11/64	14/287	0.002^c^*	6/59	5/60	0.753^c^
BMI, kg/m^2^	31.7 ± 5.3	30.1 ± 5.1	0.008^a^*	31.4 ± 5.3	31.8 ± 5.6	0.718^a^
Preoperative NRS pain in rest score	5.6 ± 2.5	4.8 ± 2.4	0.013^a^*	5.6 ± 2.5	5.3 ± 2.1	0.461^a^
Preoperative KOOS-PS score	44.4 ± 13.8	46.1 ± 13.3	0.266^a^	43.8 ± 13.6	44.5 ± 14.2	0.963^a^
Preoperative EQ-5D overall health score	69.0 ± 20.5	66.9 ± 17.2	0.056^a^	68.4 ± 20.4	66.1 ± 19.8	0.288^a^
Preoperative OKS	24.1 ± 8.6	22.8 ± 8.1	0.198^a^	24.7 ± 8.8	23.8 ± 8.2	0.531^a^

Continuous data are shown as mean ± standard deviation. Categorical data were presented as numbers and frequencies

*TKA* Total knee arthroplasty; *HTO* High tibial osteotomy; *BMI* Body Mass Index; *NRS* Numeric Rating Scale; *KOOS-PS* Knee injury and Osteoarthritis Outcome Score-Physical function Short-form; *EQ-5D* EuroQol-5 dimension; *OKS* Oxford Knee Score

^*^Statistical significance

^a^Mann-Whitney U test

^b^Independent t-test

^c^Pearson chi-square test

A comparison of patient-reported outcomes, radiological results and complication rates between TKA following HTO and primary TKA after propensity score matching is depicted in Table 2. No statistically significant between-group differences in the patient-reported outcome measures (NRS pain in rest, KOOS-PS, EQ-5D overall health score and OKS), radiographical parameters (postoperative patellar height, PTS, FTA, MPTA, aLDFA) and complication rates were found (*p* > 0.0125).

**Table 2:** Between-group comparison after propensity score matching

Outcomes	TKA following HTO (n = 65)	Primary TKA (n = 65)	*p* value
Preoperative Kellgren&Lawrence grade (III/IV)	24/41, 37%/63%	20/45, 31%/69%	0.458^c^
Preoperative patellar height (N, normality/abnormality)	58/7, 89%/11% ┼	58/7, 89%/11% ╫	1.0^c^
Postoperative patellar height (N, normality/abnormality)	58/7, 89%/11% ┼	59/6, 91%/9% ╫	0.770^c^
Preoperative PTS, degrees	87.2 ± 5.4	84.9 ± 4.7	< 0.001^a^*
Postoperative PTS, degrees	88.4 ± 2.0	89.3 ± 2.1	0.017^a^
Preoperative FTA, degrees	176.1 ± 5.1	177.5 ± 6.6	0.080^a^
Postoperative FTA, degrees	175.0 ± 3.0	175.9 ± 2.3	0.056^a^
Preoperative MPTA, degrees	91.1 ± 4.1	86.7 ± 2.4	< 0.001^a^*
Postoperative MPTA, degrees	88.2 ± 2.1	87.5 ± 1.6	0.075^a^
Preoperative aLDFA, degrees	82.5 ± 2.0	82.0 ± 2.4	0.192^a^
Postoperative aLDFA, degrees	83.4 ± 2.0	83.6 ± 2.3	0.301^a^
Postoperative NRS pain in rest 6-month, score	2.2 ± 2.2	2.2 ± 2.4	0.768^a^
Postoperative NRS pain in rest 1-year, score	2.0 ± 2.4	1.9 ± 2.3	0.934^a^
Postoperative KOOS-PS 6-month, score	29.1 ± 13.0	31.6 ± 14.9	0.347^a^
Postoperative KOOS-PS 1-year, score	29.1 ± 12.5	28.0 ± 14.8	0.651^a^
Postoperative EQ-5D health score 6-month, score	77.5 ± 15.0	74.3 ± 18.8	0.456^a^
Postoperative EQ-5D health score 1-year, score	77.2 ± 14.7	78.6 ± 14.5	0.605^a^
Postoperative OKS 6-month, score	35.6 ± 8.2	31.9 ± 10.2	0.039^a^
Postoperative OKS 1-year, score	37.2 ± 8.4	36.3 ± 9.8	0.819^a^
Complication rate	0%	1.5% ╧	1.0^d^

Continuous data are shown as mean ± standard deviation. Categorical data were presented as numbers and frequencies. The Insall-Salvati ratio defines patellar height: 0.8–1.2 (normal), > 1.2 (alta), < 0.8 (baja)

*TKA* Total knee arthroplasty; *HTO* High tibial osteotomy; *MPTA* medial proximal tibial angle; *FTA* femorotibial angle; *aLDFA* anatomical lateral distal femoral angle; *PTS* posterior tibial slope; *NRS* Numeric Rating Scale; *KOOS-PS* Knee injury and Osteoarthritis Outcome Score-Physical function Short-form; *EQ-5D* EuroQol-5 dimension; *OKS* Oxford Knee Score

^*^Statistical significance

^a^Mann-Whitney U test

^b^Independent t-test

^c^Pearson chi-square test

^d^Fisher’s exact test

┼Before TKA following HTO, seven patellar alta; After TKA following HTO, five alta, two baja

╫Before primary TKA, six patellar alta, one baja; After primary TKA, five alta, one baja

╧One had periprosthetic joint infection

The average HTO survival time in this group was 8.7 years, ranging from 1.1 to 15.1 years. In the TKA following HTO group, staples had already been removed in 46 patients (71%) before the TKA; in 19 patients, staple removal occurred during the TKA in one procedure through the midline incision. In the TKA following HTO group, 11 cases (17%) had patella resurfacing; in the primary TKA group, 15 cases (23%) had patella resurfacing.

## Discussion

The most important finding of the present study is that the patient-reported outcomes are similar between the TKA following HTO and primary TKA groups at short-term follow-ups, along with resembling between-group radiological parameters and complication rates. This finding rejects our hypothesis that patients with a TKA following HTO have inferior patient-reported outcomes compared to patients with a primary TKA.

The present study demonstrates no statistically and clinically significant disparity in postoperative patient-reported outcomes between TKA following HTO and primary TKA. This finding is consistent with previous studies when comparing patient-reported outcomes between TKA following lateral closing-wedge HTO and primary TKA: Bae et al. [9] reported no significant between-group difference in WOMAC score with a follow-up ranging from 2 to 13 years; Kazakos et al. [15] reported no significant difference in HSS with a follow-up ranging from 3 to 8 years. Other previous studies frequently used the KSS questionnaire, and no significant between-group differences in TKA following lateral closing-wedge HTO and primary TKA have been found in studies by Amendola et al. [11] and Meding et al. [6] with a follow-up ranging from 3 to 22 years. Whereas Efe et al. [7] reported a significantly lower knee score of KSS in TKA following lateral closing-wedge HTO compared to primary TKA (4–10 years follow-up), and Erak et al. [8] found higher pain levels and a significantly lower knee score of KSS in TKA following medial opening-wedge HTO compared to primary TKA (2–8 years follow-up). However, previous studies were limited by their retrospective designs and variable follow-up durations. Moreover, the evaluation of KSS is predominantly conducted from an objective standpoint by physicians, rather than relying on patient-reported assessments [16, 17]. In present study, besides no statistical significance, the between-group difference in the patient-reported outcome measures also falls below the published threshold for minimal clinically important significance [41–43]. Furthermore, our study focused on TKA following lateral closing-wedge HTO, which is dominant in our hospital and often compared in previous studies with primary TKA. There is a need for future research to focus more on the TKA following medial opening-wedge HTO.

Patients appear to exhibit similar radiological parameters in TKA following lateral closing-wedge HTO and primary TKA. The Insall-Salvati ratio is a common metric for assessing patellar height. Kazakos et al. [15] observed a significantly higher incidence of patella baja in the TKA following HTO patients than primary TKA, while Efe et al. [7] and Bae et al. [9] found no significant differences in patellar height between TKA following HTO and primary TKA. The present study showed no significant between-group difference in the incidence of abnormal patellar height, and both the TKA following HTO and primary TKA groups exhibit higher incidences of patellar alta than patellar baja following surgery. This is likely linked to the pre-existing patellar alta prior to the TKA procedures. Although the present study shows a 0.9° PTS difference between TKA following HTO and primary TKA, this disparity may be possibly explained by the measurement bias, also it lacks statistical significance and falls below the established minimal clinically significant threshold of 1.5° PTS [44]. Moreover, the TKA following HTO group exhibited a higher preoperative MPTA compared to the primary TKA group, with no significant differences in the remaining alignment parameters. This may be attributed to an observed increase in knee joint line obliquity following a valgus-producing HTO. Further research is needed to explore any lasting disparities in radiological results, including the positions of prothesis components.

Our study found no significant difference in complication rates between TKA-HTO and primary TKA. A meta-analysis showed that there was no significant difference in complication rates between TKA following HTO group and primary TKA [45], while another meta-analysis indicated a higher infection rate in TKA following HTO group compared to primary TKA [3]. Altered knee anatomy and surgical scar tissue might offer a possible explanation for the previous observations of a higher increase in infection rates among cases with a history of HTO. In our study, there was only one infection case among all analysed patients, occurring in the primary TKA group, with no significant difference in the between-group comparison of infection or complication rates. Moreover, a lateral closing-wedge HTO poses no increased risk of common peroneal nerve injury during TKA, as this nerve remains unaffected by the surgical approach for TKA. A larger patient sample may be necessary to discern the difference in complication rates between these two groups in future studies.

The present study showed a higher use of posterior-stabilized implants over cruciate-retaining ones, likely due to challenges in achieving proper tensioning of the posterior cruciate ligament in cruciate-retaining total knee arthroplasty after previous high tibial osteotomy [46]. Notably, Chen et al. [46] reported similar clinical outcomes between cruciate-retaining and posterior-stabilized implants in cases of TKA following HTO. Hence, we did not equalize the between-group distribution of implant types (cruciate-retaining and posterior-stabilized) in this study. In one case of TKA following HTO in our study, a stemmed tibial component (Legion) was chosen considering the patient’s bone quality and overweight status. Additionally, Lisy et al. [47] observed comparable patient-reported outcomes in the WOMAC scores between cruciate-retaining and kinematics-retaining implants in primary TKA.

The strength of the present study is its use of one-on-one matching, taking into account a total of eight confounding variables to mitigate their influence, thereby enhancing the robustness and validation of the evidence. Moreover, the study adopted a design with standardized follow-up length at 6-month and 1-year postoperatively. Furthermore, we employed the Bonferroni correction on the p-value to mitigate the risk of the inflation of significance, enhancing the rigor of the comparison.

A limitation of the present study was the absence of anteroposterior long-standing radiographs for patients, necessitating the use of short-knee radiographs for assessing lower limb alignment. Consequently, the femoral-tibial angle based on the anatomical axes may not serve as a reliable indicator for evaluating lower limb alignment [48]. Additionally, it is important to highlight that the study’s conclusions were derived from a relatively short follow-up period, underscoring the necessity for future studies with extended follow-up durations.

## Conclusions

The short-term assessment of TKA following HTO indicates outcomes similar to primary TKA. A previous HTO does not impact the early results of subsequent TKA, suggesting that the previous HTO has minimal influence on TKA outcomes.
